# Acupuncture accelerates wound healing via CGRP-RAMP1-TSP1-mediated macrophage M2 polarization

**DOI:** 10.1186/s13020-025-01255-2

**Published:** 2025-11-18

**Authors:** Xiaoer Liu, Junsheng Chen, Lingyue Zou, Xiaohan Lu, Boran Zhu, Jingwen Li, Yuyan Zhu, Minjiao Jiang, Rou Peng, Yifan Guo, Shengfeng Lu

**Affiliations:** 1 Key Laboratory of Acupuncture and Medicine Research of Ministry of Education, Nanjing University of Chinese Medicine, Nanjing, 210023 China; 2 School of Elderly Care Services and Management, School of Aging Industry, Nanjing University of Chinese Medicine, Nanjing, 210023 China; 3 Jiangsu Province Engineering Research Center of TCM Intelligence Health Service, Nanjing University of Chinese Medicine, Nanjing, 210023 China

**Keywords:** Acupuncture, CGRP, Wound healing, Inflammation, Macrophage

## Abstract

**Background:**

Macrophages orchestrate the immune microenvironment during skin wound healing. While acupuncture’s efficacy in accelerating wound healing is established, its underlying mechanisms, particularly those related to macrophage modulation, remain poorly characterized. This study aimed to investigate how acupuncture modulates macrophage phenotype and inflammatory responses to facilitate skin repair.

**Methods:**

We established an 8-mm full-thickness dorsal skin defect model in C57BL/6 J mice, randomizing them into control and acupuncture groups. To investigate the role of the calcitonin gene-related peptide (CGRP) pathway, the CGRP receptor antagonist BIBN4096 was administered intradermally before each acupuncture treatment. For the acupuncture group, we performed a daily 20-min intervention for 10 days, which consisted of oblique manual needling at four predefined locations around the wound. Wound repair quality, inflammatory cytokine levels, and macrophage polarization were assessed using histological analysis (H&E and Masson's staining), flow cytometry, enzyme-linked immunosorbent assay (ELISA), immunofluorescence, and reverse transcription quantitative polymerase chain reaction (RT-qPCR).

**Results:**

Acupuncture significantly facilitated wound closure, enhanced collagen deposition, and improved tissue repair quality. These benefits were associated with an immunomodulatory effect, characterized by enhanced M2 macrophage polarization within the wounds, a reduction in systemic macrophage load in the spleen, and reduced local and systemic levels of IL-1β and IL-6. Mechanistically, the activation of the CGRP-RAMP1-TSP-1 pathway was critical, as its inhibition with BIBN4096 abrogated the effects of acupuncture on macrophage polarization and wound healing. Notably, the suppression of inflammatory cytokines by acupuncture was only partially dependent on CGRP signaling.

**Conclusions:**

Our findings indicate that acupuncture promotes wound healing and inflammation resolution, at least in part, by activating the CGRP-RAMP1-TSP-1 pathway to drive M2 macrophage polarization. Furthermore, the persistence of its anti-inflammatory effects after CGRP inhibition strongly suggests the involvement of additional, non-CGRP-dependent pathways in modulating the immune response.

**Graphical Abstract:**

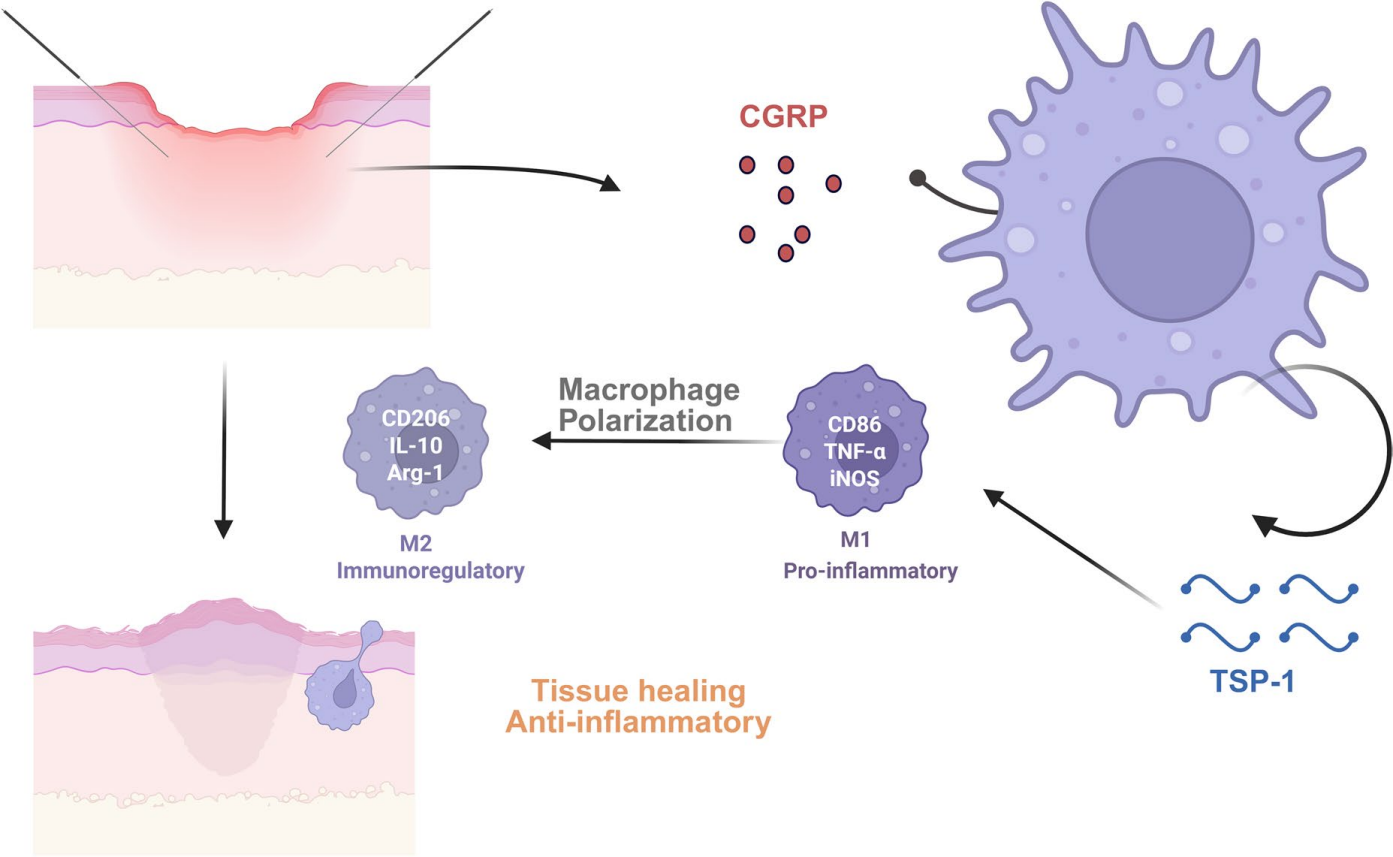

## Introduction

The skin, the body's largest organ, forms the principal protective barrier against external threats, including microbiological, mechanical, chemical, osmotic, and thermal hazards [1]. Disruption of this barrier by trauma, lacerations, incisions, or contusions initiates a wound-healing program classically partitioned into overlapping inflammatory, proliferation, and remodeling phases [2]. Early inflammation enhances antimicrobial defense, whereas subsequent pro-reparative responses drive tissue regeneration [3]. Owing to their remarkable plasticity and persistence within inflammatory infiltrates, macrophages are central regulators of the cutaneous immune milieu [4]. In response to cytokine signals, they adopt pro-inflammatory (M1) or anti-inflammatory (M2) phenotypes [5]. M1 macrophages predominantly facilitate early wound closure by augmenting phagocytic activity and secreting pro-inflammatory cytokines (e.g., IL-6, IL-12, TNF-α) to eliminate pathogens and cellular debris [6]. By approximately day 5 post-injury, M2 macrophages typically replace M1 populations, dampening inflammation and releasing fibrotic and angiogenic mediators to promote tissue restoration [7]. Thus, the timely M1-to-M2 transition is a key determinant of healing efficiency and quality. Acupuncture, a cornerstone of traditional Chinese medicine, has demonstrated therapeutic efficacy across diverse pathologies, including analgesia [8], immunomodulation [9], and accelerated wound repair [10]. Peri-wound acupuncture promotes healing through dual mechanisms, suppressing inflammatory cytokines and enhancing angiogenesis [10]. Critically, acupuncture exerts both local and systemic effects by regulating the neuro-endocrine-immune (NEI) network, which integrates neural, endocrine, and immune pathways to maintain homeostasis and support self-repair [11]. Sensory neurons and cutaneous cells communicate via neuropeptides (e.g., neurohormones, neurotrophins) and their receptors, with calcitonin gene-related peptide (CGRP) playing a particularly critical role. CGRP facilitates fibroblast proliferation, resolves neurogenic inflammation, and stimulates epidermal regeneration via keratinocyte activation [12].

Emerging evidence indicates that CGRP modulates immune responses during wound healing by interacting with receptor activity-modifying protein 1 (RAMP1) on neutrophils, monocytes, and macrophages. This interaction promotes macrophage polarization toward anti-inflammatory/pro-reparative phenotypes by inducing thrombospondin-1 (TSP-1), a multifunctional extracellular matrix protein with endocrine/paracrine effects [13]. Acupuncture has been shown to upregulate CGRP expression in multiple tissues, including the gut, hypothalamus [14], spinal cord [15], and skin [16, 17]. However, the precise role of CGRP in acupuncture-accelerated wound healing remains incompletely understood.

To address this knowledge gap, we employed a murine model with deep skin injuries to investigate how acupuncture shapes local inflammation and the behavior of immune cells. Using CGRP receptor inhibitors, we further elucidated the specific mechanistic contributions of CGRP. Our findings provide compelling evidence that the NEI network plays a pivotal role in mediating acupuncture-induced wound healing through its integrated regulation of neural, endocrine, and immune components.

## Material and methods

### Animals

Male C57BL/6 mice, aged 7 weeks and weighing 21–23 g, were purchased from Shanghai Slaccas Co. Ltd. (License No.: SCXK(Shanghai)2022–0004). The Ethics Committee of Animal Experimentation at Nanjing University of Chinese Medicine authorized the study protocol (approval number: 202409A074). The Specific Pathogen-Free (SPF) animal facility at Nanjing University of Chinese Medicine housed the mice. All experimental techniques were performed in compliance with the National Institutes of Health Guide for the Care and Use of Laboratory Animals. The mice were acclimatized and nourished for a week before the experiment. The temperature was regulated between 20 and 25 °C, while the humidity was controlled between 40 and 45%. The light/dark cycle lasted 12 h each, and the mice had unrestricted access to food and water.

### Animal groups

Male C57BL/6 mice were randomly placed into different groups: control group, model group, acupuncture group, model + saline group, acupuncture + saline group, model + BIBN4096 group, and acupuncture + BIBN4096. The BIBN4096 group of mice received an intradermal injection of the CGRP receptor inhibitor BIBN4096 (Selleck, S8212, dissolved in saline, concentration 1 mg/kg) surrounding the lesion [18]. The control group was administered an equivalent dosage of saline via the same injection method. All mice received either saline or BIBN4096 injections one hour before the acupuncture procedure to ensure adequate time for the drug to take effect.

### Animal models

This study constructed a whole-layer damage wound model using mouse dorsal skin for experimentation [19]. The murine model for skin damage was developed as previously described, and is widely adopted in wound-healing studies. In summary, mouse hair removal and skin preparation were conducted the day prior to the experiment, followed by the treatment of skin damage on the day of the procedure. Prior to the experiment, a razor was employed to eliminate the majority of the hair on the mice's backs, followed by the application of depilatory cream to eradicate the remaining clipped hair. The application duration of the depilatory cream was restricted to 30 s to prevent chemical burns and discomfort resulting from an extended skin healing process. The region was subsequently cleansed with an alcohol pad to eliminate the surplus depilatory cream. The process was conducted on a heated pad to prevent a decrease in mouse body temperature caused by alcohol evaporation. Prior to modeling, prepare the recovery cage by spraying and washing it with 75% ethanol, then disinfect the autoclaved cage, ensuring that gauze pads are also included within it. Subsequently, prepare 5–10 g of breeding food and add 5–10 drops of water to the food. Prepare water bottles with elongated spouts. The surgical wound was found at the raised area on the mouse's back, where an 8 mm hole punch was used to gently press the skin, making a circular mark, and then sterilized surgical scissors were used to cut around that mark. The mice were kept in separate cages after modeling to prevent mutual wound disruption. Every two days thereafter, we documented the mouse wounds using a stationary camera position and measured them with a ruler. This dorsal full-thickness excisional wound model provides the advantages of standardization, reproducibility, and suitability for dynamic monitoring of wound healing. However, it also has limitations, including differences between murine and human skin healing and limited capacity to mimic chronic or infected wounds.

### Acupuncture treatment

The intervention was performed using acupuncture needles surrounding the wound. First, 5% isoflurane was used for general anesthesia, followed by 2% isoflurane for maintenance anesthesia at a flow rate of 0.3–0.6 L/minute. Mice in the treatment group were obliquely punctured using 0.18*13 mm acupuncture needles (Hwato, 20,162,270,970, Suzhou, China) at the 3, 6, 9, and 12 o'clock positions around the wounds, following a protocol based on previously described peri-wound acupuncture techniques in skin injury models [10, 20]. Manual acupuncture treatment was administered for a total of 20 min, with stimulation every five minutes. It involved thrusting and lifting as well as twisting and rotating 120 times per minute (about 2 Hz), with each manipulation lasting one minute. This treatment regimen was conducted daily over a period of 10 days. The manipulation was executed by the same proficient and certified acupuncturist.

### HE staining and Masson staining

After dehydration, transparency, and embedding by classical procedures, skin tissue was cut into 4 μm thick sections with a microtome (LEICA RM2016) and placed on a glass slide. Paraffin sections were dewaxed and rehydrated by xylene and alcohol. For HE staining, the cell nuclei of the sections were stained blue with hematoxylin and the cytoplasm was stained red with eosin staining solution. For Masson staining, sections were made red with Ponceau acid fuchsin solution for muscle fibers and blue with aniline blue for collagen fibers. All sections were observed under a Leica Thunder microscope and images were collected, and the images were counted using imageJ software.

### Immunofluorescence staining

Following the mice’s harvest, the back skin tissue was left in a 4% formaldehyde solution for the whole night. To achieve total dehydration, the tissue was incubated overnight at 4 °C in a 20% sucrose solution containing 0.1% PBS on the same day. The tissue was then embedded in the OCT embedding media. Sections were produced at −20 °C and placed on slides with a thickness of 7 μm. The sections were stained with collagen I (Affinity, AF7001), arginase-1 (CST, AF7001), iNOS (CST, 13120S), CGRP (Abcam, ab81887), Ramp1 (Proteintech, 10,327–1-AP), TSP-1 (Abcam, AB267388), and F4/80 (Huabio, RT1212). The corresponding secondary antibodies, Alexa Fluor 594 (Abcam: AB150080), Alexa Fluor 488 (Abcam: AB15013), and goat anti-rat IgG (H + L) labeled with fluorescein isothiocyanate (FITC) (Proteintech, SA00003-11), were then incubated at 37 °C for one hour after the primary antibodies were added dropwise and incubated overnight at 4 °C. A confocal microscope (Leica Thunder, Germany) was then used to obtain fluorescent pictures after the samples had been treated with an anti-fluorescence quenching blocker containing DAPI and covered with a coverslip.

### Flow cytometry

Mouse spleen tissue was extracted in a six-well plate containing PBS, mechanically homogenized using a needle tail, and subsequently filtered through a strainer. The resulting suspension was centrifuged at 1500 rpm for 5 min at 4℃. Subsequently, the spleen samples underwent lysis with 800 μl of erythrocyte lysate for 5–10 min at 4℃ in subdued lighting, followed by two washes with PBS. The cells were then resuspended and analyzed in separate tubes. The following delineates the surface staining protocol for macrophage and neutrophil cells: Incubate with CD16/32 antibody (eBioscience 14–0161-82) at a concentration of 0.5 μl per tube for 5 min at 4 ℃. Next, introduce the antibody mixture (macrophage: F4/80-FITC (eBioscience, 11–4801-82), CD11b-APC (eBioscience, 17–0112-83), CD206-PE (eBio Invitrogen 12–2061-82); neutrophil: CD11b-APC, LY6G-PE) and incubate for 40 min in darkness. All samples were washed with PBS and then resuspended in 300 μl of PBS. The samples were fixed overnight with 4% paraformaldehyde. The samples were filtered via a 70 μm filter and subsequently analyzed with a flow cytometer (Beckman Coulter, Moflo XDP) before machine operation, with a blank tube and a single reference tube prepared for calibration. The experimental data were examined with FlowJo software for circular gating.

### Enzyme-Linked immunosorbent analysis

After serum collection, whole blood is centrifuged at 3000 × g for 15 min at 4 °C after being allowed to clot for 15 min at room temperature. The material is kept at − 80 °C and the supernatant is disposed of. The sample is centrifuged at 12,000 × g for 15 min at 4 °C after skin tissue has been weighed and homogenized using ice-cold tissue lysis buffer. The supernatant is then discarded and stored at − 80 °C. With absorbance determined at 450 nm within 15 min, all tests were conducted using the mouse IL-1β ELISA kit (Jing Yibai, LA128804H, Nanjing, China) and the mouse IL-6 ELISA kit (Jing Yibai, LA128802H, Nanjing, China).

### Western blot

After removing 30 mg of skin tissue using sterile surgical scissors, add 300 μL of protease and phosphatase inhibitors. Grind until all of the tissue has been broken up. Use the BCA technique to measure the protein content after centrifuging the protein lysate for 10 min at 12,000 rpm and 4 °C. Then, move the supernatant to an EP tube. Proteins were transferred to a PVDF membrane after being separated by electrophoresis. After that, the membrane was blocked for an hour by incubating it in TBST buffer that contained 5% non-fat milk. Primary antibodies were then incubated on the membrane. CGRP (Abcam, ab81887), Ramp1 (Proteintech, 10,327–1-AP), TSP-1 (Abcam, AB267388), GAPDH (Proteintech, 60,004–1-LG), and β-actin (Affinity, AF7018) were the antibodies that were utilized. The membrane was diluted 1:1000 and incubated for the entire night at 4 °C. The membrane was then treated for two hours at 25 °C with the secondary antibody (Proteinintech, SA00001-2, 1:5000). The ratio of the target protein bands' gray values to those of the β-actin and GAPDH bands was used to calculate the target proteins' relative expression levels.

### RT-qPCR

Total RNA was isolated utilizing the RNA Extraction Kit (Vazyme RT101). Total RNA was extracted using the manufacturer's protocol, and the RNA concentration was quantified. Subsequently, reverse transcription was conducted under the specified conditions: 37 degrees Celsius for 15 min and 85 degrees Celsius for 5 s. Homogeneously blended materials were placed into the wells of a 384-well plate, and the PCR reaction was performed under the following conditions: Denaturation at 95 °C for 5 min; 95 °C for 10 s; 60 °C for 60 s, repeated for 40 cycles. Gene sequences were obtained from the National Center for Biotechnology Information (NCBI) database, and gene-specific primers were designed and validated using Primer Premier. The primers were manufactured by Shanghai Shengong Bioengineering Technology Service Co. The specific primers used in this process are listed in the accompanying Table 1.

**Table 1:** Primer sequence

Gene	Forward sequence	Reverse sequence
β-actin	CAGGCGGTGCCTATGTCTC	CCTGGCACCCAGCACAAT
TNF-α	GGGCCGGACTCGTCATAC	CTTGTCGATGAGGAACTGTGGAGAG
IL-10	TACTTCTCCCGGGATGACGT	GTCCTGTTCCGGCTTCTTGA
CD86	TCAATGGGACTGCATATCTGCC	GCCAAAATACTACCAGCTCACT
CD206	GACGCTCTAAGTGCCATCTC	ATAACTCTGTGCCCTTGATTCC
CGRP	CAGTGCCTTTGAGGTCAATCT	CCAGCAGGCGAACTTCTTCTT
Ramp1	GACGCTATGGTGTGACT	GAGTGCAGTCATGAGCAG
TSP-1	GCAACCGCATTCCAGAGTC	GGGGCTGGATAGATCTTGGC

### Statistical analysis

The experimental data were analyzed using SPSS 24.0 statistical software, with the results expressed as mean ± SD. A t-test was utilized to assess differences between the model group and the acupuncture group, whilst one-way ANOVA was applied for comparisons among several groups. A P value of less than 0.05 was considered statistically significant.

## Result

### Acupuncture accelerates and enhances the quality of wound healing

**Fig. 1:**
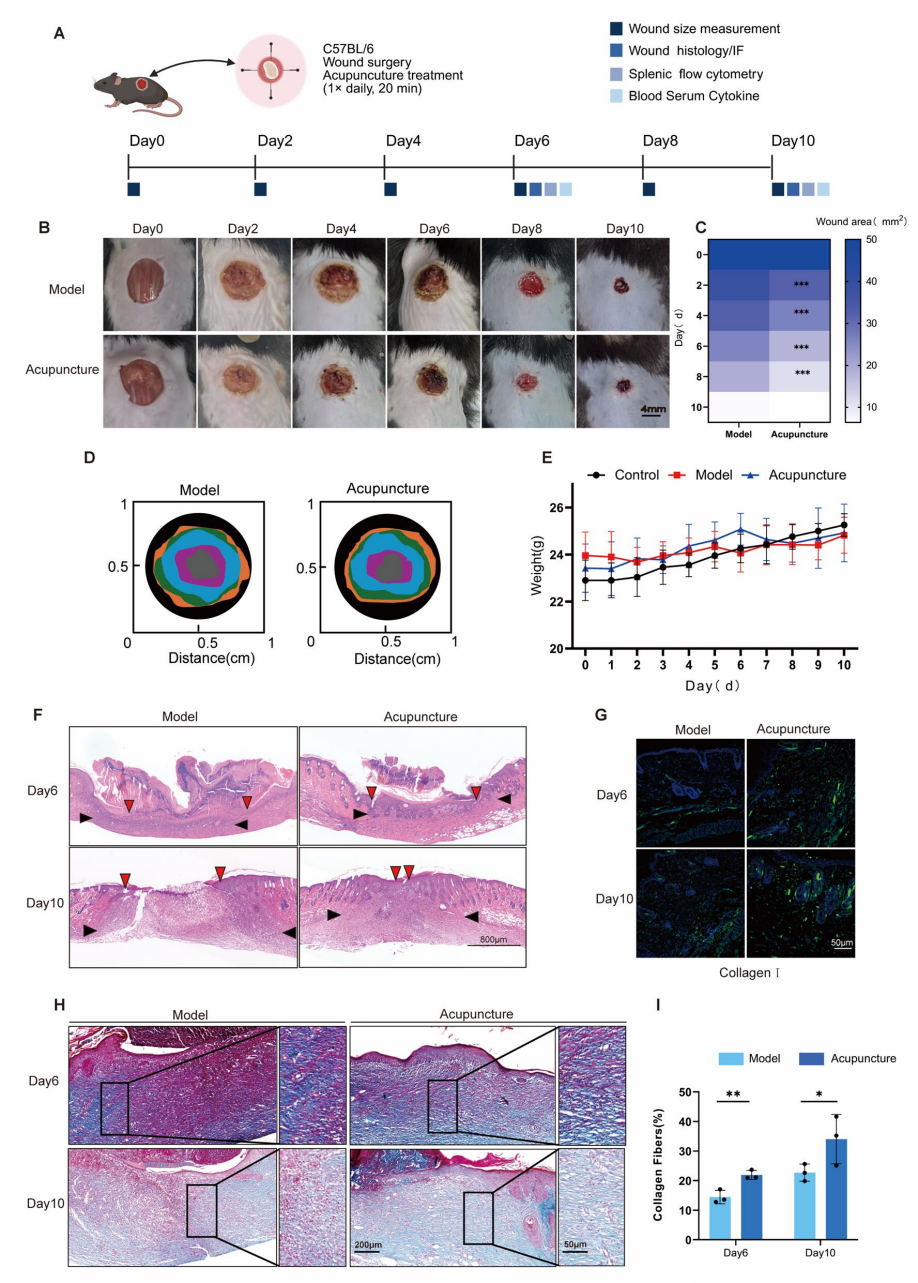
Acupuncture expedites wound healing and improves the overall quality of the healing process. **A** The animal flow diagram depicts the temporal schedule of the C57BL/6 mice skin injury model experiment. The experiment involves performing dorsal wound surgery on the mice on Day 0 and administering hand needling around the wound for 20 min every day after that date. The horizontal axis of the image represents the pivotal time intervals (Day 2, Day 4, Day 6, Day 8, Day 10) from the initiation of the experiment (Day 0) to Day 10. The subsequent experimental procedures were conducted at various intervals: quantifying the wound dimensions, microscopically evaluating the wounds, analyzing splenic cells, and assessing the blood for cytokines. **B** Photographic documentation chart for skin wounds. Scale bar = 4 mm. Two groups share scale bars. **C** Statistical variations in the domain of dermatological injuries. n = 5; *P < 0.05; **P < 0.01; ***P < 0.001. **D** Simulations of mouse wounds at various temporal intervals. **E** Graphs depicting alterations in body weight across three groups of mice at various time intervals. n = 5. **F** Wound closure was assessed using histomorphometric analysis on days 6 and 10 post-injury. The red arrows denote the boundaries of the epidermis, whereas the black arrows signify the boundaries of the epithelial ligule. Scale bar = 800 μm. **G** An immunofluorescence experiment was conducted on two groups of mice on Day 6 and Day 10 to evaluate tissue collagen **Ⅰ**. n = 3. **H**, **E** Masson staining outcomes of wound tissues on days 6 and 10, accompanied by statistical graphs. Day 6, n = 3; Day 10, n = 3; *P < 0.05; **P < 0.01; ***P < 0.001

To investigate the effects of local acupuncture on tissue healing, we created an 8-mm-diameter circular wound on the back of mice and performed acupuncture around the wound for 10 days, starting on the first day after wound creation (Fig. 1A). We evaluated the wound size on days 0, 2, 4, 6, 8, and 10 and recorded its macroscopic appearance using a fixed-position camera (Fig. 1B). Through statistical analysis, we found that beginning on the second day of treatment, the wound area in the acupuncture group was notably smaller than in the control group. Additionally, we observed no significant change in body weight (Fig. 1C-E). Although there was no significant difference in wound area between the two groups on day 10 after skin injury, hematoxylin and eosin (HE) staining revealed that acupuncture significantly promoted wound epithelium regeneration (Fig. 1F). Furthermore, immunofluorescence and Masson staining revealed that the acupuncture group had a higher proportion of type I collagen in their wounds and that the collagen fibers were more closely arranged (Fig. 1G-I). Together, these results suggest that acupuncture accelerates skin wound healing and improves the quality of skin injury repair.

### Acupuncture attenuates the systemic inflammatory response

**Fig. 2:**
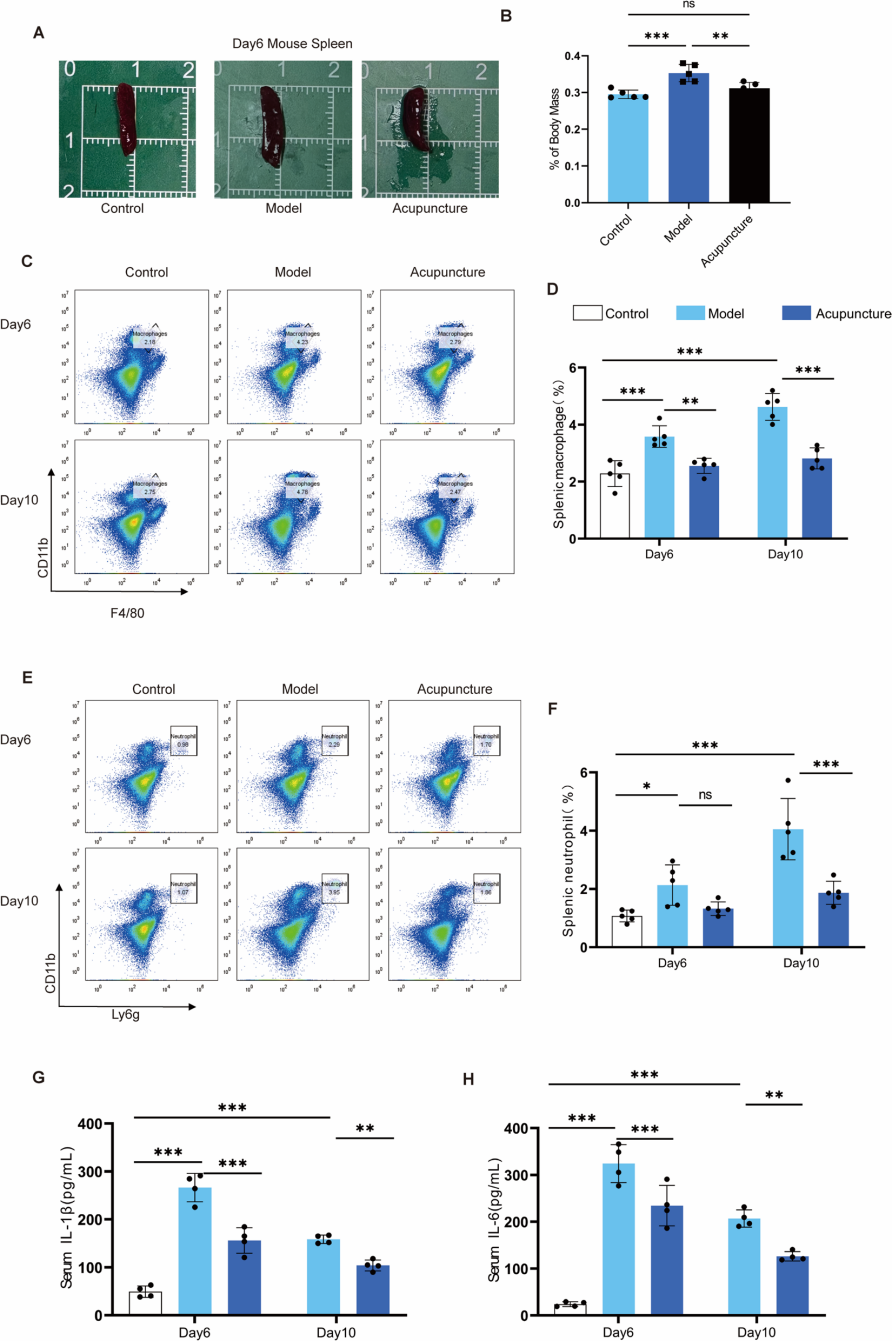
Acupuncture improves systemic inflammation levels and affects spleen immune levels. **A** Photo-recorded graphs of spleen size of mice in three groups of control, model, and acupuncture groups with uniform scale size in the background. n = 5. **B** Spleen/body weight ratios of mice in three groups: control, model, and acupuncture. n = 5; *P < 0.05; **P < 0.01; ***P < 0.001. **C**, **D** Spleen on days 6 and 10 in the control, model, and acupuncture groups Macrophage flow cytometry scatter plots as well as statistical graphs of cell-circle-gate results. n = 5; *P < 0.05; **P < 0.01; ***P < 0.001. **E**, **F** Flow cytometry scatter plots of splenic neutrophils on days 6 and 10 in the control, model, and acupuncture groups, as well as statistical graphs of cell-circle-gate results. n = 5; *P < 0.05; **P < 0.01; ***P < 0.001. **G**, **H** ELISA assay for IL-1β as well as IL-6 in serum on days 6 and 10 in the control, model, and acupuncture groups. Day6, n = 4, Day10, n = 4, *P < 0.05; **P < 0.01; ***P < 0.001

To investigate the mechanism by which acupuncture promotes wound healing, this study first evaluated its effects on systemic immune responses. As a core immune organ, the spleen plays a crucial role in maintaining immune homeostasis by recruiting and generating immune cells to regulate both local and systemic immune responses. The spleen-to-body-weight ratio serves as an indicator reflecting the degree of systemic immune activation [21]. Experimental results showed that, compared to the control group, mouse spleen volume and the spleen/body weight ratio significantly increased on day 6 after skin injury modeling. However, acupuncture treatment for 6 days effectively reversed these changes (Fig. 2A-B). Macrophages and neutrophils in the spleen are key cell types that reflect systemic inflammatory status [22]. Flow cytometry analysis was performed to detect the proportions of CD11b + F4/80 + macrophages and CD11b + Ly6g + neutrophils in the spleen. As expected, the full-thickness skin incision model significantly increased the percentage of macrophages and neutrophils in the spleen compared to the control group (Fig. 2C-F). Acupuncture intervention significantly reduced the proportion of both cell types on days 6 and 10, indicating that acupuncture can suppress trauma-induced systemic immune activation sustainably (Fig. 2C-F). Furthermore, serum levels of the proinflammatory cytokines IL-1β and IL-6 supported these findings (Fig. 2G-H). Taken together, these data demonstrate that acupuncture effectively attenuates systemic inflammatory responses in mice with skin injury.

### Acupuncture attenuates local inflammation in injured skin and induces macrophage M2 polarization

**Fig. 3:**
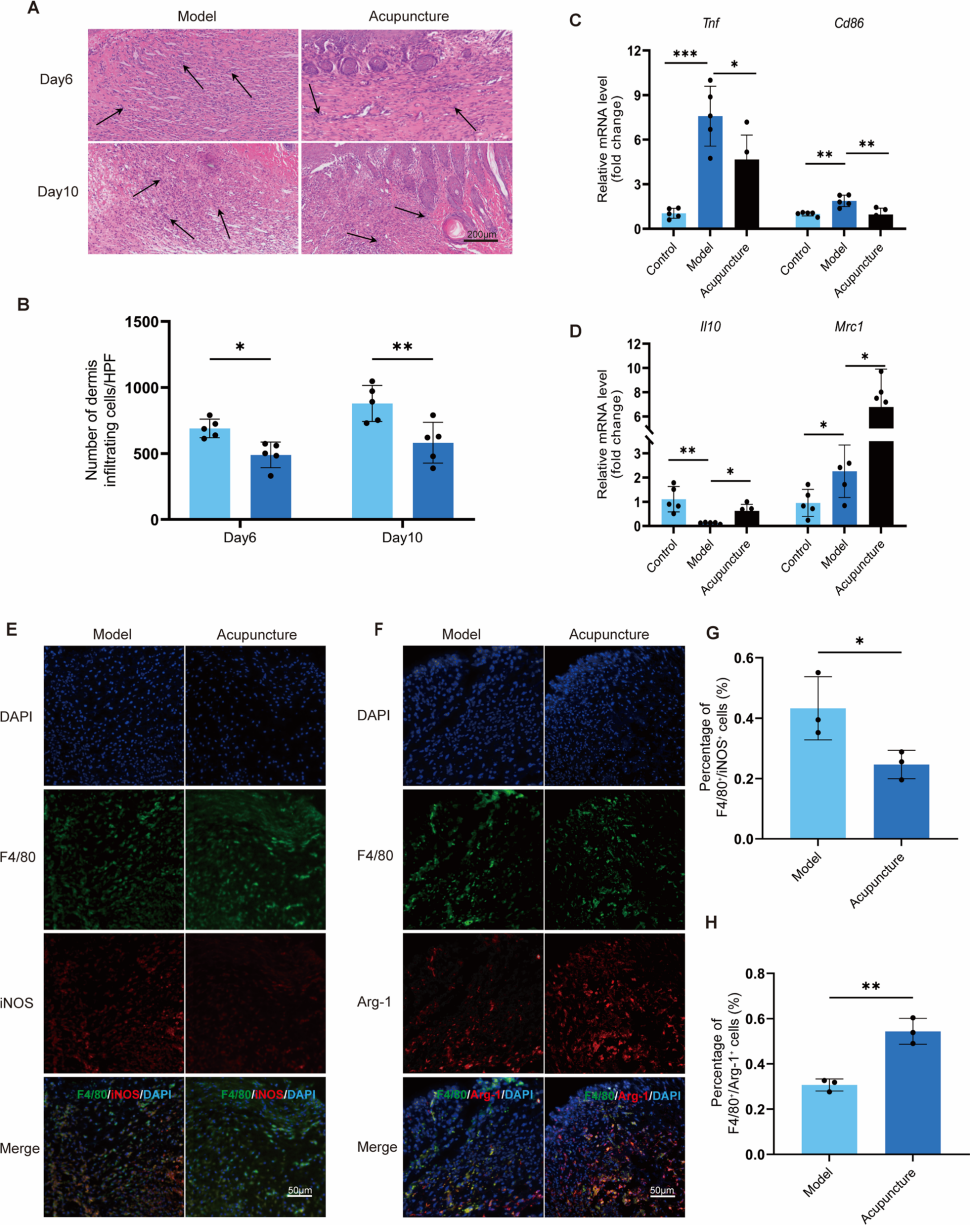
Acupuncture diminishes inflammation in local tissues and expedites local macrophage polarization. **A**, **B** High magnification microscopic images of skin tissues from the model and needling groups were taken on days 6 and 10, and the analysis of monocyte infiltration was performed. Scale bar = 200 μm; n = 3; *P < 0.05; **P < 0.01; ***P < 0.001. **C**, **D** The mRNA levels of TNF-α and CD86, which indicate M1 macrophages, as well as IL-10 and CD206, which indicate M2 macrophages in local tissues, were measured. n = 5; *P < 0.05; **P < 0.01; ***P < 0.001. **E**, **F** The results of the macrophage immunofluorescence in local skin tissues used F4/80 (green) to identify all macrophages, while iNOS (red) and Arg-1 (red) were used to identify M1 and M2 macrophages. Scale bar = 50 μm, n = 3. **G**, **H** The quantitative statistics of immunofluorescence staining findings for co-labeling F4/80 and iNOS positive cells were enumerated for M1 macrophages, while F4/80 and Arg-1 positive cells were marked for M2 macrophages. n = 3; *P < 0.05; **P < 0.01; ***P < 0.001

To investigate the effect of acupuncture on the local inflammatory response in injured skin, we first assessed immune cell infiltration by H&E staining and quantified mononuclear cells in the dermis (indicated by black arrows). The results showed that acupuncture treatment significantly reduced immune cell infiltration in the injured skin tissue on both day 6 and day 10 (Fig. 3A-B). Given the critical role of macrophage phenotype regulation in injury repair, we further examined the effect of acupuncture on macrophage polarization. Macrophages exhibit remarkable plasticity and are dynamically regulated by microenvironmental signals, generally classified into the pro-inflammatory M1 phenotype (marked by CD86, TNF-α, iNOS, etc.) and the reparative M2 phenotype (marked by CD206, IL-10, Arg1, etc.). qPCR analysis revealed that acupuncture significantly downregulated mRNA expression of TNF-α and CD86, while simultaneously upregulating the expression of IL-10 and CD206 in the local injured skin (Fig. 3C-D). To further validate these findings, we performed immunofluorescence staining using F4/80 as a pan-macrophage marker, iNOS for M1 macrophages, and Arg-1 for M2 macrophages (Fig. 3E-F). The results demonstrated that, compared to the model group, the acupuncture group showed a significantly lower proportion of M1 macrophages (F4/80⁺ iNOS⁺) (Fig. 3G) and a significantly higher proportion of M2 macrophages (F4/80⁺ Arg-1⁺) (Fig. 3H), indicating that acupuncture promotes local macrophage polarization toward the M2 phenotype. In summary, our study demonstrates that acupuncture effectively attenuates local inflammation in injured skin, which may be associated with its ability to induce macrophage M2 polarization.

### Acupuncture activated the CGRP/Ramp1/TSP-1 pathway in skin wounds

**Fig. 4:**
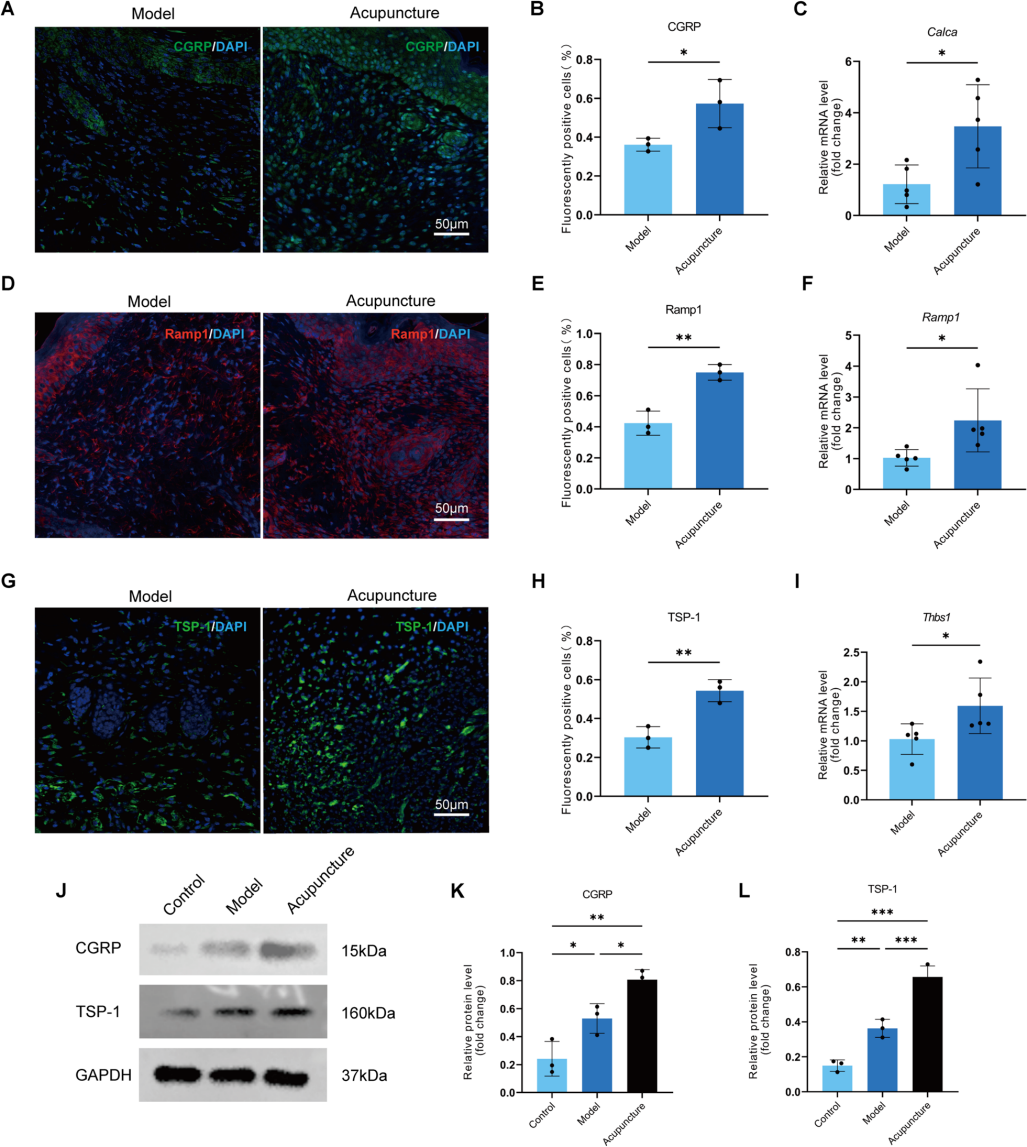
Increase in the expression of the acupuncture-activated CGRP/Ramp1/TSP-1 pathway. **A**, **D**, **G** On the sixth day of localized wound tissue, the immunofluorescence of CGRP, Ramp1, and TSP-1 shows that CGRP and TSP-1 are green, Ramp-1 is red, and Dapi is blue. Scale bar = 50 μm, n = 3. **B**, **E**, **H** The number of CGRP/Ramp1/TSP-1-positive cells was determined by counting the immunofluorescence results. n = 3; *p < 0.05, **p < 0.01, ***P < 0.001. **C**, **F**, **I** CGRP, Ramp1, and TSP-1 mRNA levels in local wound tissues on day six. n = 5; *p < 0.05, **p < 0.01, ***P < 0.001. **J** On day six, the three groups—all of which were chosen using GAPDH as an internal reference—showed protein expression levels of CGRP and TSP-1. The molecular weights of CGRP and TSP-1 were 15 kDa and 160 kDa. n = 3. M Ramp1 protein expression levels, molecular weight of 33 kDa, and internal reference β-actin were chosen. n = 3. **K**, **L**, N The results of the CGRP/Ramp1/TSP-1 protein expression level were quantified using respectively. n = 3, *p < 0.05, **p < 0.01, ***P < 0.001

CGRP regulates inflammatory responses and tissue healing through complex neuro-immune connections. Recent studies have reported that the CGRP/Ramp1/TSP-1 signaling pathway plays a significant role in the post-wound healing process of the skin, accompanied by changes in the phenotype of macrophages [13]. Given that acupuncture can upregulate the expression of CGRP in various tissues, we hypothesized that acupuncture promotes local wound healing through the CGRP/Ramp1/TSP-1 signaling pathway. On the 6th day after trauma, we obtained skin tissue samples from mice and conducted tests. The results showed that acupuncture significantly increased the positive staining and mRNA levels of CGRP and its receptor RAMP1 in the skin trauma tissue (Fig. 4A-F). As expected, the expression of TSP-1 in the local skin of the acupuncture group was also upregulated synchronously (Fig. 4G-I). To further confirm whether CGRP and TSP-1 changed at the protein level, we performed Western blot (WB) detection. The results showed that acupuncture could further upregulate the expression of CGRP and TSP-1 (Fig. 4J-L). These results suggest that acupuncture can activate the CGRP-Ramp1-TSP-1 pathway in skin wounds, which may be the molecular mechanism by which it accelerates wound healing.

### CGRP receptor antagonism impairs acupuncture-accelerated wound healing

**Fig. 5:**
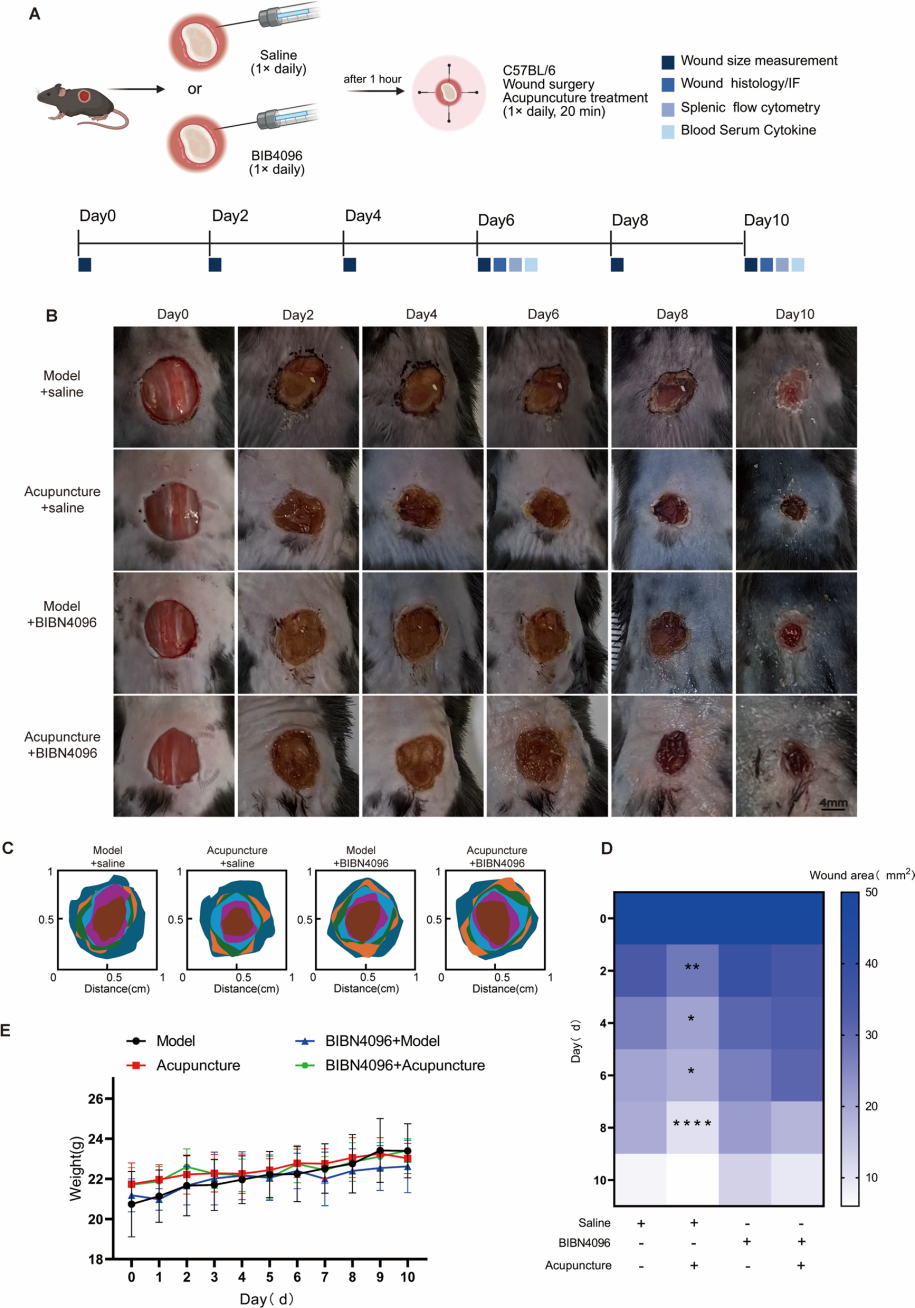
BIBN4096 inhibits the wound-healing effects of acupuncture. **A** Schematic representation of the experimental protocol: following traumatic surgery on C57BL/6 mice, subjects received daily treatments of acupuncture and either saline or BIBN4096 injections, subsequently evaluated for traumatic histology/immunofluorescence (IF), splenic flow cytometry, and serum cytokines at specified time intervals. **B** Wound healing was documented in the model + saline group, the acupuncture + saline group, the model + BIBN4096 group, and the Acupuncture + BIBN4096 group. Scale bar = 4 mm; n = 5. **C** Quantification of wound area distribution among several treatment groups at each radial distance from the wound region. **D** Dynamic thermogram of the wound area, with color intensity representing the size of the wound and asterisks denoting statistical differences between groups. n = 5; *p < 0.05, **p < 0.01, ***P < 0.001. **E** Mouse body weight change curve (days 0–10): illustrating the impact of various treatments on the overall physiological condition of the mice. n = 5

**Fig. 6:**
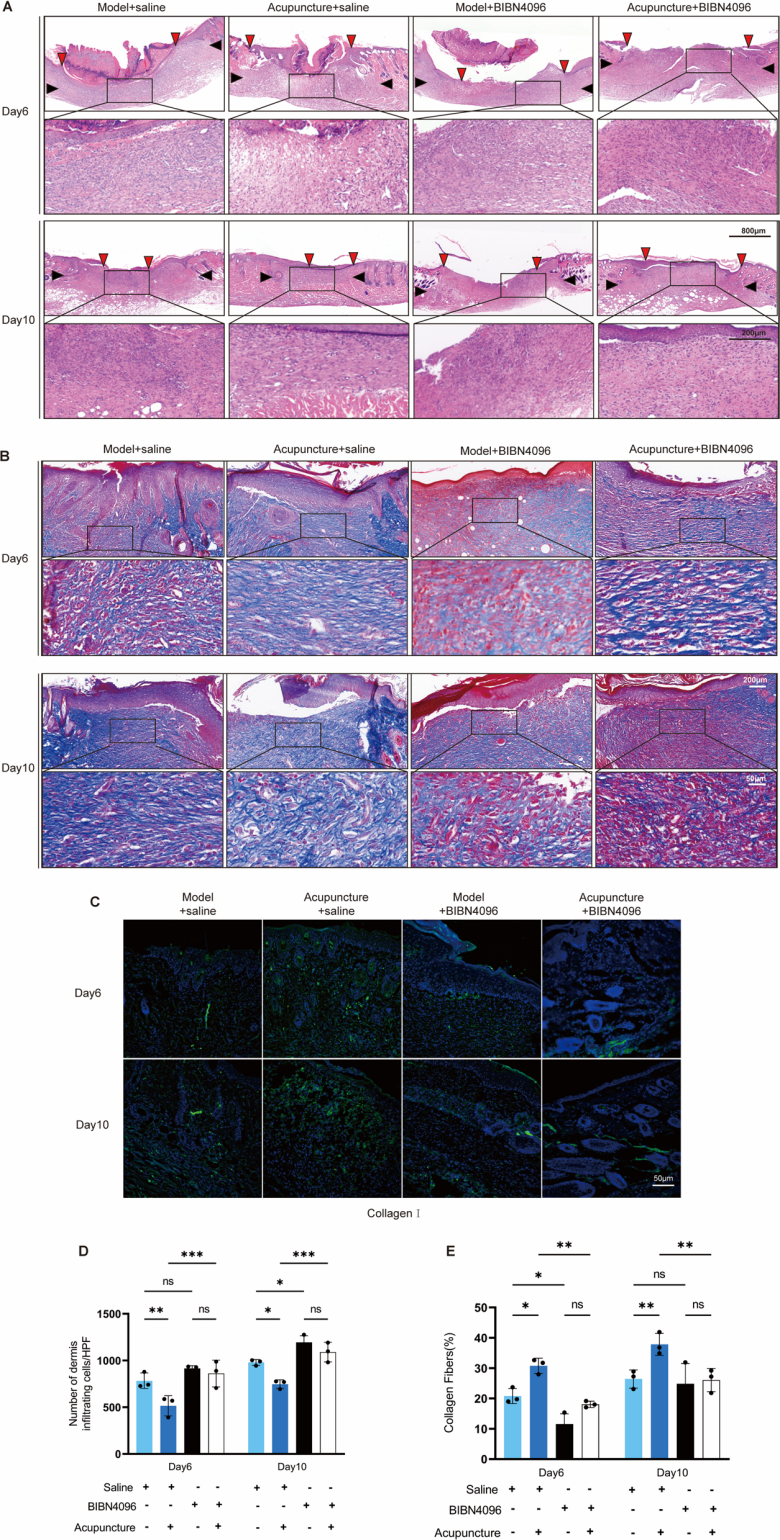
BIBN4096 blocks acupuncture's enhancement of wound healing quality. **A** Four sets of H&E-stained sections are presented. The upper section displays Day 6, while the lower section exhibits Day 10. Scale = 800 μm (panoramic view); scale = 200 μm (fine view); n = 3. **B** Masson stained sections. The upper section depicts Day 6, while the lower section illustrates Day 10, highlighting the collagen remodeling process. Scale = 200 μm (panoramic view); scale = 50 μm (fine view); n = 3. **C** Immunofluorescence maps illustrated the temporal distribution characteristics of Collagen I in the wound on days 6 and 10. Scale = 50 μm; n = 3. **D** Results of quantifying monocyte infiltration in the four groups individually. n = 3, *p < 0.05, **p < 0.01, ***P < 0.001. **E** Results of collagen area quantified via Masson staining. n = 3; *p < 0.05, **p < 0.01, ***p < 0.001

To further verify the role of the CGRP/Ramp1/TSP-1 pathway in acupuncture-accelerated wound healing, we administered intradermal injections of the CGRP receptor antagonist (BIBN4096) around the wound margins (Fig. 5A). Analysis of wound area quantification revealed that wound closure was significantly delayed in the BIBN4096-treated group compared to the saline-injected control group (Fig. 5B-D). No significant difference in body weight was observed among the groups during this period (Fig. 5E). Consistently, histological examination by H&E staining demonstrated that BIBN4096 administration attenuated the pro-healing effects of acupuncture, manifesting as delayed re-epithelialization, reduced hair follicle neogenesis, diminished tissue regeneration, and an increased infiltration of inflammatory cells in the dermis at both day 6 and day 10. (Fig. 6A and 6D). Furthermore, Masson's trichrome staining and collagen I immunofluorescence staining indicated that BIBN4096 completely abrogated the beneficial effects of acupuncture on healing, resulting in diminished collagen deposition and delayed skin regeneration (Fig. 6B, C, E). These collective findings confirm that pharmacological blockade of the CGRP receptor impedes cutaneous wound healing and compromises healing quality.

### CGRP receptor antagonism inhibits acupuncture-induced TSP-1 expression and M2 macrophage polarization

**Fig. 7:**
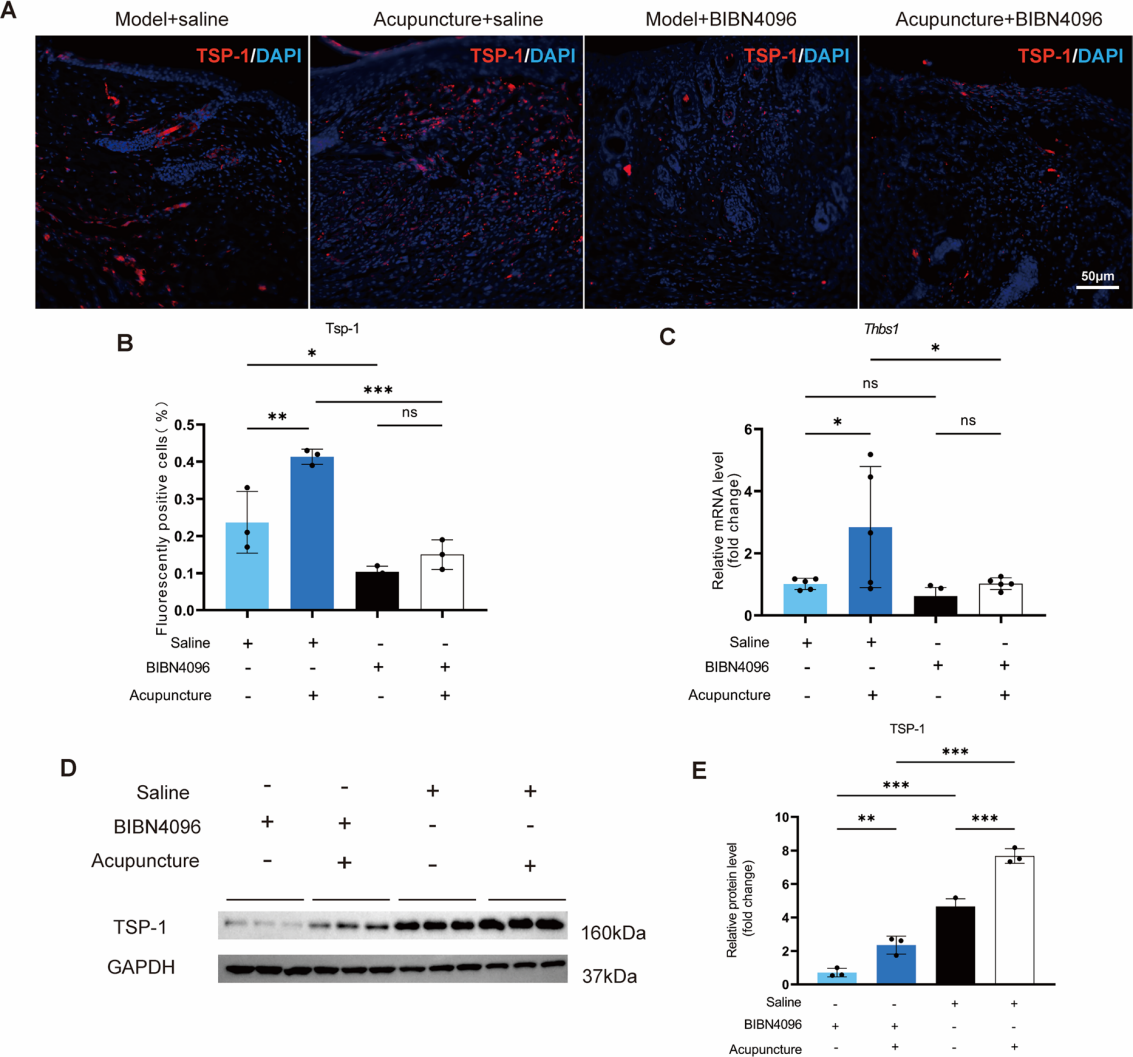

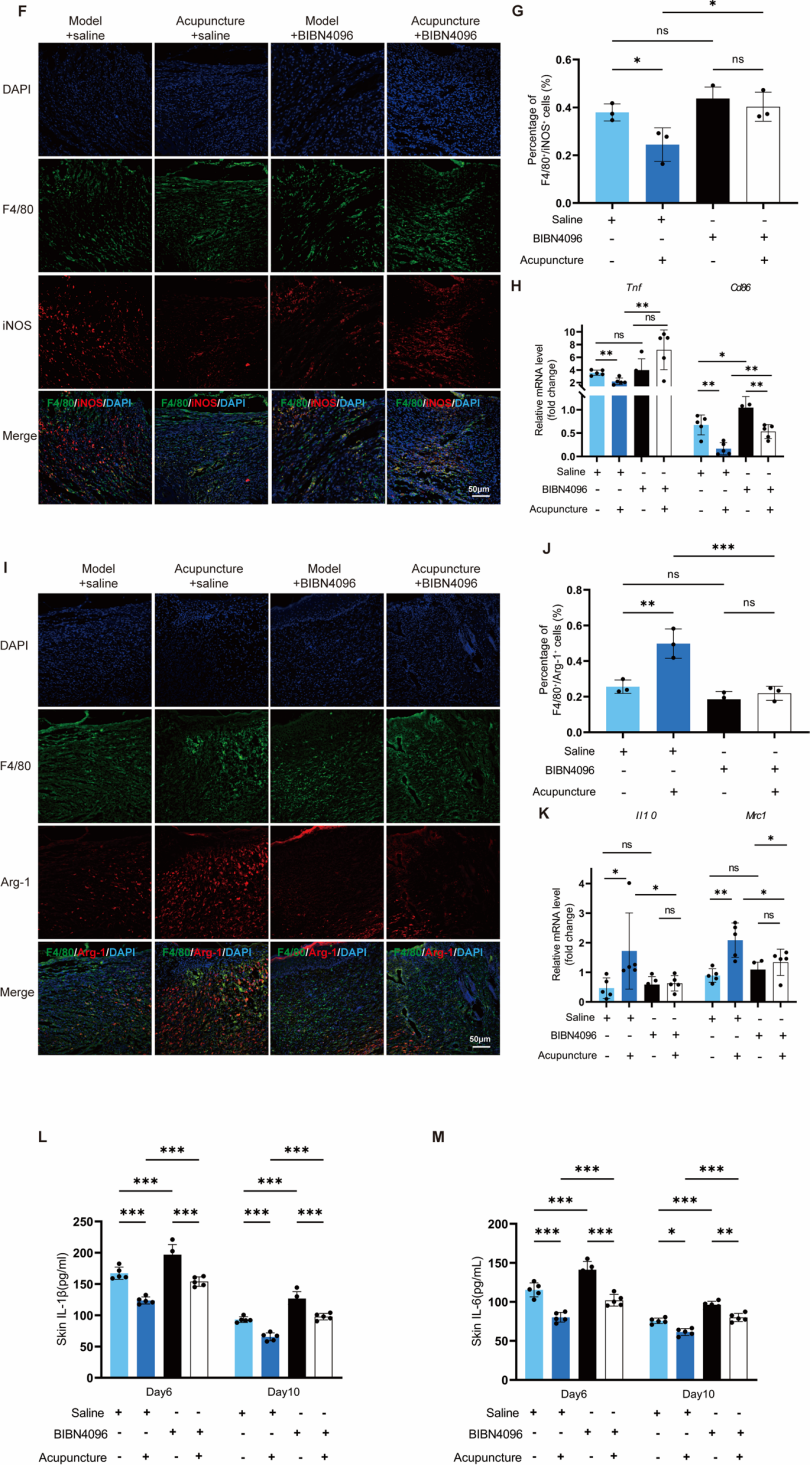
BIBN4096 obstructs the acupuncture-induced increase in TSP-1 expression, hence reducing the anti-inflammatory effects of acupuncture. **A**, **B** Prior to the injection of saline into the initial two groups, BIBN4096 was administered to the subsequent two groups. TSP-1 expression in local tissues was assessed via immunofluorescence, and the percentage of TSP-1 positive cells was measured. Scale = 50 μm; n = 3; *p < 0.05, **p < 0.01, ***p < 0.001. **C** The mRNA expression of TSP-1 in the local tissues following the administration of saline and BIBN4096. n = 5; *p < 0.05, **p < 0.01, ***p < 0.001. **D**, **E** Protein expression levels in local tissues were assessed using GAPDH as an internal reference, with TSP-1 exhibiting a molecular weight of 160 kDa and GAPDH 37 kDa, followed by quantitative analysis. n = 3; *p < 0.05; **p < 0.01; ***p < 0.001. **F**, **J** ELISA analysis of IL-1β and IL-6 concentrations in localized wound tissue. n = 5; *p < 0.05; **p < 0.01; ***p < 0.001. **H**, **I** Immunofluorescence analysis of,cell markers was conducted on local tissues, with DAPI-stained nuclei appearing blue. Notable co-localization was observed for the macrophage marker F4/80 (green) and the pro-inflammatory marker iNOS (red). The percentage of positively stained cells for both markers was quantitatively assessed. Scale bar = 50 μm; n = 3; *p < 0.05, **p < 0.01, ***p < 0.001. **J** Quantitative mRNA values for TNF-α and CD86 in localized skin tissues. n = 5; *p < 0.05, **p < 0.01, ***p < 0.001. **K**, **L** Conducted immunofluorescence on localized tissues to identify markers of M2 cells, utilizing DAPI for blue labeling of nuclei, the macrophage marker F4/80 in green, and the inflammation suppression marker Arg-1 in red. Quantitative analysis of significant co-localization results was conducted to determine the percentage of positive cells for both markers. Scale bar = 50 μm; n = 3; *p < 0.05, **p < 0.01, ***p < 0.001. **M** Quantitative mRNA analysis of localized tissue IL-10 and CD206. n = 5; *p < 0.05, **p < 0.01, ***p < 0.001

Immunofluorescence results revealed that acupuncture significantly increased TSP-1 (red) positive staining in the saline group, whereas BIBN4096 impeded the acupuncture-induced upregulation of TSP-1 expression (Fig. 7A-B). This conclusion was further confirmed by RT-qPCR and WB results (Fig. 7C-E). The expression of TSP-1 influences macrophage migration and polarization. We observed that BIBN4096 blocked the ability of acupuncture to downregulate M1 markers (iNOS, TNF-α, and CD86) in skin wounds (Fig. 7F-H), while also inhibiting acupuncture-induced upregulation of M2 markers (Arg-1, IL-10, and Mrc1) (Fig. 7I-K). These results indicate that the CGRP receptor antagonist hinders acupuncture-driven M2 polarization of macrophages in skin wounds. Notably, although BIBN4096 significantly elevated levels of the local pro-inflammatory factors IL-1β and IL-6 in the skin, the anti-inflammatory effects of acupuncture were not completely abolished (Fig. 7L-M). This finding suggests that, beyond activating the CGRP-RAMP1-TSP-1 pathway to induce M2 macrophage polarization, acupuncture may regulate the local inflammatory microenvironment through alternative mechanisms.

### CGRP signaling mediates the systemic anti-inflammatory effects of acupuncture

**Fig. 8:**
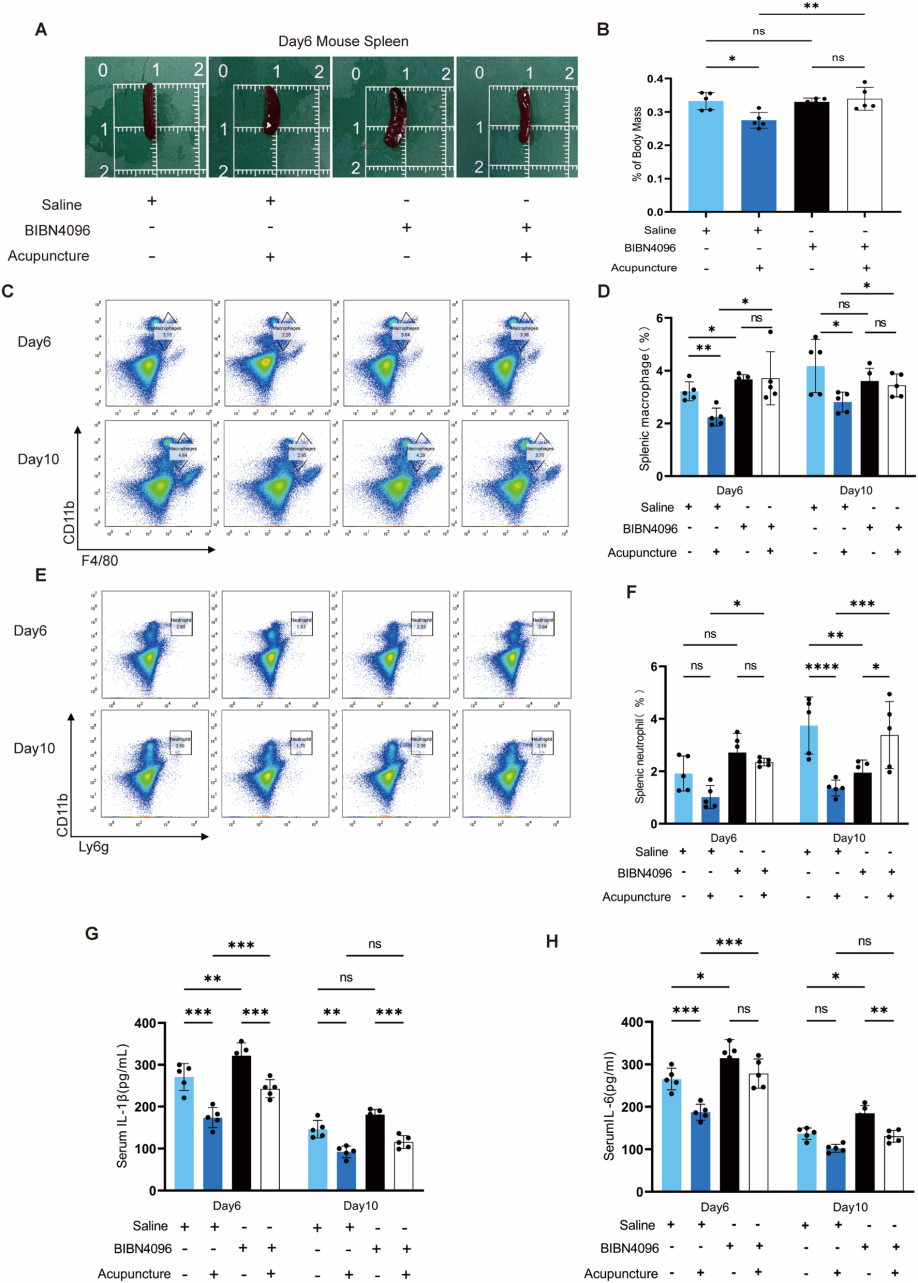
BIBN4096 can diminish the systemic inflammatory symptoms associated with acupuncture. **A** Representative spleen size graph for four mouse groups at day 6. **B** Statistics for the spleen body weight ratio. n = 5; *p < 0.05, **p < 0.01, ***p < 0.001. **C**, **E** Splenic flow cytometry scatter plots, use flow cytometry to resolve alterations in splenic immune cell populations during wound healing. **D**, **F** The proportions of CD11b-positive Ly6G neutrophils and F4/80 macrophages in spleen were statistically detected on days 6 and 10. n = 5; *p < 0.05, **p < 0.01, ***p < 0.001. **G**, **F** Serum proinflammatory factor levels of IL-1β and IL-6 as determined by ELISA. n = 5; *p < 0.05, **p < 0.01, ***p < 0.001

Based on our previous findings that acupuncture improves systemic and local inflammation and that BIBN4096 antagonizes its effect on local inflammation, we evaluated the role of the CGRP-RAMP1-TSP-1 pathway in the systemic anti-inflammatory effects of acupuncture by locally injecting BIBN4096 intradermally. Our study revealed that BIBN4096 did not alter the baseline spleen-to-body weight ratio but significantly blocked the acupuncture-induced reduction in this ratio (Fig. 8A-B). These results suggest that CGRP signaling plays a crucial role in the regulation of systemic inflammation by acupuncture. Flow cytometry analysis of the spleen showed that BIBN4096 increased the proportion of macrophages on day 6 post-injury, but not on day 10. More importantly, BIBN4096 completely blocked the acupuncture-induced reduction in the proportion of macrophages on days 6 and 10 (Fig. 8C-D).

However, BIBN4096 exhibited a time-dependent, paradoxical effect on neutrophils. On day 6, there was a non-significant trend toward an increased neutrophil proportion in the BIBN4096 + acupuncture group compared to the saline + acupuncture group (Fig. 8E-F). By day 10, however, BIBN4096 itself significantly reduced the neutrophil proportion. It unexpectedly reversed the effect of acupuncture, resulting in a significantly higher neutrophil proportion in the BIBN4096 + acupuncture group (Fig. 8E-F). This contradictory phenomenon likely stems from the dynamic nature of the inflammatory response. CGRP may exert distinctly different immunomodulatory effects at these stages by regulating neutrophil recruitment, apoptosis, or clearance.

To further investigate the systemic inflammatory status, we measured blood inflammatory cytokines. On day 6, BIBN4096 significantly increased IL-1β and IL-6 levels and partially antagonized the effects of acupuncture; it blocked the acupuncture-induced reduction in IL-6 but did not affect the IL-1β level (Fig. 8G-H). By day 10, BIBN4096 still elevated baseline IL-6 levels, but it no longer affected the reduction of IL-1β and IL-6 induced by acupuncture (Fig. 8G-H). This temporal and cell-subpopulation-specific regulation of cytokines reveals the complexity of CGRP's role in the neuro-immune axis. It may simultaneously modulate inflammatory mediators from different sources and dynamically interact with other pathways activated by acupuncture, such as the cholinergic anti-inflammatory pathway

.

## Discussion

This study is the first to confirm that acupuncture promotes M2 polarization of macrophages and improves the local inflammatory microenvironment by activating the CGRP-RAMP1-TSP1 pathway, thereby accelerating skin wound healing and tissue remodeling. These findings offer crucial molecular insights into the neuroimmune mechanisms underlying acupuncture-mediated tissue repair.

Acupuncture, a well-established traditional therapy, has been reported to promote skin wound healing through modalities such as manual acupuncture [10] and electroacupuncture [20]. However, the underlying molecular mechanisms remain incompletely elucidated. In this study, we demonstrate that acupuncture not only accelerated early wound closure (Day 6) but also enhanced the quality of late-stage healing (Day 10). These improvements were characterized by augmented collagen deposition and a restructured, more organized fibrotic architecture, collectively indicating an abbreviated healing cycle and a superior repair outcome.

These improvements were driven by precise immunomodulation. Acupuncture significantly attenuated both systemic and local inflammation, as demonstrated by reductions in systemic inflammatory markers, diminished splenic macrophage infiltration, and a shift in skin macrophages toward the M2 phenotype. Macrophages play a central role in skin repair. Whereas M1 macrophages dominate the inflammatory phase to clear debris and combat infection, their persistent activity impedes healing. The transition to M2 polarization represents a critical switch that promotes extracellular matrix (ECM) production and re-epithelialization. By day 10, this immunomodulatory shift had not only resolved inflammation but also established a favorable microenvironment conducive to collagen remodeling and organized fibrotic structure formation, which ultimately enhanced the quality of tissue regeneration [23].

M2 macrophages are widely recognized as pro-repair cells that express a range of healing-related factors, including arginase-1, ECM components, and growth factors such as VEGF-A, PDGF, and IGF [24]. However, the mechanisms guiding macrophage polarization toward a reparative phenotype remain incompletely understood [25]. Emerging evidence suggests that the neuropeptide CGRP, which signals through RAMP1, plays a role in modulating immune cell function during wound healing. CGRP influences neutrophils, monocytes, and macrophages by inhibiting recruitment, promoting apoptosis, enhancing efferocytosis, and inducing macrophage polarization toward a pro-repair phenotype [13]. These effects are partly mediated through the induction of TSP-1 and its autocrine/paracrine actions. Macrophages represent a major source of TSP-1 in wounds, and TSP-1 deficiency in these cells has been linked to impaired healing and sustained inflammation [26]. TSP-1 is an important regulator of immune responses [27]. In the presence of anti-inflammatory cytokines such as IL-4, IL-13, or IL-10, it enhances the expression of CD206 and arginase-1. This action promotes the shift of macrophages toward an anti-inflammatory, pro-repair phenotype.

To determine whether the CGRP-RAMP1-TSP1 pathway functionally contributes to healing, we blocked CGRP signaling using the antagonist olcegepant (BIBN-4096) [28, 29]. The resultant blockade suppressed TSP-1 expression, delayed wound closure, compromised tissue repair, and reduced M2 polarization. These findings demonstrate that this pathway is necessary for mediating the pro-healing benefits of acupuncture, likely by driving local macrophage phenotypic switching. Notably, while CGRP blockade abolished the reduction in splenic macrophage numbers, the downregulation of serum inflammatory factors persisted, implying that CGRP-independent pathways contribute to the systemic anti-inflammatory actions of acupuncture. In addition to the local CGRP-RAMP1-TSP1 axis, acupuncture may transmit somatic signals to the central nervous system, activating multiple regions ranging from the spinal cord to the brainstem (including the nucleus tractus solitarius, rostral ventromedial medulla, and dorsal motor nucleus of the vagus) and hypothalamus. This central processing activates multiple efferent pathways—including the vagus-adrenal-dopamine axis [30], sympathetic pathways [31], and the HPA axis [32]—that systemically modulate immune activity via neurotransmitters and hormones. For example, the cholinergic anti-inflammatory pathway fine-tunes key intracellular signaling cascades such as JAK2/STAT3, NF-κB, and MAPK to promote inflammation resolution [33]. The integrated engagement of these multimodal mechanisms may explain how acupuncture sustains its systemic anti-inflammatory effect even under CGRP pathway blockade.

The neuroimmunomodulatory functions of the CGRP-RAMP1-TSP1 axis can be pharmacologically mimicked using CGRP receptor agonists or TSP-1 activators [34, 35]. However, the clinical translation of CGRP-based therapies faces challenges related to peptide stability, controlled release, and systemic side effects. Moreover, such pharmacological strategies lack the dynamic, feedback-responsive regulation inherent to neural stimulation. In contrast, acupuncture operates through multi-level neural circuits, enabling coordinated regulation of inflammation and tissue repair with minimal cost and invasiveness. Nevertheless, its broader clinical adoption is limited by variability in technique and patient acceptance. Given these complementary profiles, combining acupuncture with targeted pharmacological agonists may be a promising integrated strategy to enhance wound healing outcomes.

We acknowledge that this study has several limitations. First, the role of the CGRP-RAMP1-TSP1 axis in non-macrophage lineages (e.g., neutrophils) was not examined. Second, the mechanism behind the reduction in splenic macrophages—whether due to apoptosis, inhibited egress, or other processes—remains unresolved and requires further study.

In summary, our study elucidates a novel mechanism by which acupuncture promotes wound repair via the CGRP-RAMP1-TSP1 pathway, coordinating local and systemic immune responses. This work advances our understanding of acupuncture's neuroimmunomodulatory properties and opens new avenues for treating refractory chronic wounds. Integrating traditional acupuncture with contemporary neuroimmune insights may foster the development of precision treatment strategies in regenerative medicine.

## Data Availability

All data are available upon request from the corresponding author.

## Abbreviations

Arg-1: Arginase-1
CD11b: Cluster of differentiation 11b
CD16/32: Cluster of differentiation 16 and 32
CD206: Cluster of differentiation 206
CD86: Cluster of differentiation 86
CGRP: Calcitonin gene-related peptide
DRG: Dorsal root ganglion
ECM: Extracellular matrix
F4/80: EGF-like module-containing mucin-like hormone receptor-like 1
HPA axis: Hypothalamic-pituitary-adrenal axis
IGF: Insulin-like growth factor
IL-10: Interleukin-10
IL-1β: Interleukin-1 beta
IL-6: Interleukin-6
iNOS: Inducible nitric oxide synthase
JAK2: Janus kinase 2
LY6G: Lymphocyte antigen 6 complex locus G6D
M1: Classically activated (pro-inflammatory) macrophage
M2: Alternatively activated (anti-inflammatory) macrophage
MAPK: Mitogen-activated protein kinase
NEI: Neuro-endocrine-immune
NF-κB: Nuclear factor kappa-light-chain-enhancer of activated B cells
PDGF: Platelet-derived growth factor
RAMP1: Receptor activity-modifying protein 1
STAT3: Signal transducer and activator of transcription 3
TNF-α: Tumor necrosis factor alpha
TSP-1: Thrombospondin-1
VEGF: Vascular endothelial growth factor
